# What These Findings Tell Us. Reply to Kelly et al. What Do These Findings Tell Us? Comment on “Tinella et al. Cognitive Efficiency and Fitness-to-Drive along the Lifespan: The Mediation Effect of Visuospatial Transformations. *Brain Sci.* 2021, *11*, 1028”

**DOI:** 10.3390/brainsci12020178

**Published:** 2022-01-29

**Authors:** Luigi Tinella, Antonella Lopez, Alessandro Oronzo Caffò, Francesco Nardulli, Ignazio Grattagliano, Andrea Bosco

**Affiliations:** 1 Department of Educational Sciences, Psychology, Communication, University of Bari, 70121 Bari, Italy; antonella.lopez@uniba.it (A.L.); alessandro.caffo@uniba.it (A.O.C.); ignazio.grattagliano@uniba.it (I.G.); andrea.bosco@uniba.it (A.B.); 2 Commissione Medica Locale Patenti Speciali, Azienda Sanitaria Locale-Bari, 70121 Bari, Italy; francesco.nardulli@asl.bari.it

**Keywords:** path analysis, regression models, participant selection, Montreal cognitive assessment (MoCA), spatial cognition, fitness to drive

## Abstract

The study of the contribution of spatial transformation skills to driving behavior is a research topic substantiated by scarce evidence. In previous studies, we found that mental rotation and perspective-taking skills have an influence on performance in driving tasks by conveying the distal effects of the general cognitive efficiency on the execution of driving maneuvers. Studies have provided evidence on the relevance of the cognitive processes of encoding, imagined rotation, and spatial orientation in the accuracy of both the vehicle management during stressful driving situations and the acquisition of visual information on the traffic scenario. Results can find applications in both the training and the assessment of fitness to drive, as well as in the study of interaction between the drivers and in-vehicle devices. The lack of cross-validations in path analysis models cannot be assumed, a priori, to be capitalizing on chance and as an example of bad science. The non-replicability of a study should be demonstrated before it is proclaimed. The purpose of this reply was to address the questions raised by Kelly et al. (2022)—that is, “Do these results seem replicable?” and “How do these results advance our understanding of brain function and/or human behavior?”—by providing additional information on the study in question.

## 1. Introduction

The studies commented upon by Kelly et al. (2022) [1] presented important similarities because they are both part of the same PhD research project of the first author (L.T.). However, it was not completely clear if the received comment was addressed only to the more recent study, or if it encompasses both of those called into question. Anyway, some crucial aspects that structurally differentiated these studies should be noted.

The above-mentioned project deals with the study of cognitive (i.e., mostly spatial cognitive functions) and personality determinants of fitness to drive (FtD). The project also includes an investigation of clinical samples that are not the object of the commented-upon study/studies. Both studies employed the same measures of general cognitive status (MoCA), mental rotation (MRT), perspective taking (PT), and the prerequisites of fitness to drive (reaction times, resilience of attention, and perceptual speed, as part of the Vienna Test System–DRIVESC). The earlier work [2] investigated the influence of the MoCA, MRT, and PT (as predictors) on driving measures (as outcomes) in a sample of young and adult drivers (aged from 18 to 64). Age, gender, and years of education were controlled in the analysis. The effects of the first-order interactions between demographic and cognitive variables on measures of FtD were also assessed in this study.

Instead, the second work [3] examined the structural relationships among the same variables in a sample of male participants aged from 18 to 91. A mediated mediation model of relationships between variables was tested to find out a more efficient explanation of the results found in the first study. In both studies, participants belonged to two different data collection processes with an estimated overlap of about 26% of male participants.

The purpose of this report was to address the questions raised by Kelly et al. (2022) [1]: “Do these results seem replicable?” and “How do these results advance our understanding of brain function and/or human behavior?”

## 2. Do These Results Seem Replicable?

The term HARKing (hypothesizing after the results are known) generally indicates the strategy of presenting an a posteriori hypothesis as if it were predetermined [4]. In his recent work, Mark Rubin [5] (p. 308) describes three types of HARKing: “(1) using current results to construct post hoc hypotheses that are then reported as if they were a priori hypotheses; (2) retrieving hypotheses from a post hoc literature search and reporting them as a priori hypotheses; and (3) failing to report a priori hypotheses that are unsupported by the current results”.

Following these definitions, the first type of HARKing practice assumes that hypotheses must be constructed when results are well known. In our publication history, the results of the first study became the starting point of the aim formulated in the second study (to explore structural relationships among the same variables in a sample involving also older participants), so no a posteriori hypotheses were stated.

The second HARKing practice assumes that hypotheses must been formulated by retrieving them from an a posteriori bibliographic search. To the best of our knowledge, the study in question [3] is the first to explore pathways among the predictors of driving fitness by including measures of both mental rotation and perspective-taking skills. The research topic investigating the contribution of spatial imagined transformations (and representations) on driving behaviors is quite new (until now it “*has been neglected*” in driving research; see for instance [6] (p. 260)). In conclusion, neither the first nor the second type of Harking can be applied to our publication story.

The third kind of HARKing practice involves the omission of predetermined hypothesis that are unsupported by the obtained results. This practice implies that all the obtained results, or at least most of them, should have a perfect fit with the predetermined hypotheses. Conversely, few predetermined hypotheses reported in the study in question have been only partially supported by results. Therefore, in that study, not all hypotheses formulated have been corroborated by the results. In particular, (a) the mediated mediation of both the spatial skills was hypothesized to predict the resilience of attention and perceptual speed. Conversely, only mental rotation predicted the resilience of attention, while only perspective taking predicted the perceptual speed, both through single mediations. Moreover, it was hypothesized that (b) mental rotation would have showed a mediation role between the cognitive functioning and the reaction speed. Again, this hypothesis was not verified by the results. Finally, hypotheses on both (c) the negative effect of age on driving measures and (d) the positive effect of the MoCA expected on resilience of attention and perceptual speed only were formulated following results obtained in our first study, and they have been verified even in the second study. In this case, it is unclear how we would have practiced this third kind of HARKing, since only two out of four among the declared hypotheses have been found.

According to Kelly et al., the studies “lack important details concerning selection of study participants, leading to puzzling discrepancies in the participant selection criteria for the two studies”. However, (a) the assumptions underlying each study differed, (b) the two studies concerned diverse age cohorts, and (c) there were different data collections at the base of the two studies (the enrolment criteria from the first to the second study were partially modified). In both the studies, participants were required to (i) have Italian as their mother tongue, (ii) have a valid current driving license, provisional or above, (iii) have normal or corrected vision, and (iv) not being or had been a professional driver. The first study included five enrolment criteria, while the second had six. Both the studies were limited to a sample of (v) active drivers; however, while the former work included participants who have driven at least one time in the previous month, the second study included those who reported driving at least once in the previous week. These more restrictive constraints justify why the second paper involves only a quarter of the participants of study one. This discrepancy was the consequence of the different aims of the studies. The first study was completely explorative, while the second represented an attempt to find a more efficient and exhaustive explanation from the point of view of the structural relationships between variables through pathways models. The relationships between variables which emerged firstly in terms of statistical causal models, if further confirmed by studies of other groups/centers, might provide suggestions on the brain processes and on the circuits involved in supporting driving performance. To address the aim of the second study, we have chosen to limit the investigation to those drivers with a stronger driving experience, i.e., those most frequently exposed to driving activity in everyday life. As stated above, the sample of the second study involved only male participants aged from 18 to 91 years. The samples of the two studies differed because the second included only males and older drivers.

The group of women drivers was excluded in the second study, since few of them (adult and older) met the enrolment criteria. The same reason explains why three male participants were excluded. Anyway, most of female drivers included in the first study reported driving two or three times in the previous month. This may be the consequence of the cultural trends in driving activity for older women in our geographic/cultural area. Despite the continuous increase in the number of older women behind the wheel [7], they represent a minority when compared to older male drivers (i.e., age 65–74: M, 81%; F, 42%; age 75+: M, 61%; F, 18%; SafetyNet 2009) especially in the south of Italy (the place in which the studies were carried out). Women tend to reduce their driving activity during ageing, until definitively avoiding driving [8,9,10].

Nevertheless, the decision to restrict the investigation to male drivers was made to avoid discrepancies between (1) the gender group sizes and (2) the distribution of age within the gender groups. In other words, this was an attempt to avoid capitalizing on the previous data collection. Finally, the fact that a “convenience sample” was included in the second study had been clearly reported as a limitation in the paper.

To consider active drivers, those who drive at least once a week or once a month (or in the last week/last month) is a well-consolidated stance in driving research [11,12,13]. Furthermore, in the second study, the MoCA total score inclusion cut-off above 17 was added among criteria to include only participants who were in (vi) a general healthy cognitive state based on the Italian cut-off. Table 1 shows the participant selection criteria for both the studies.

Four out of the five enrolment criteria were the same among the studies. Considering the methodological reasons supporting the choice, to both modify and add an extra criterion in the second study, such discrepancies may perhaps seem less “puzzling” now.

Kelly and colleagues pointed out some statistical limitations regarding path analyses, structural equation modelling, and factor analyses. These issues limit the replicability of the results in path analysis as, potentially, in any multivariate statistical technique. We are also aware of solutions such as that indicated in the commentary, and that the sample size does not always allow for cross-validations. The suggestions described by Kelly and colleagues are useful and widely used methods to avoid these statistical limitations. Even in this case, this issue was listed among the limitations, where the need to replicate the studied model on a larger and more representative sample of drivers was clearly pointed out. With an awareness of the limitations due to statistics-related methods, and in turn to the generalization of results, the study provided tentative evidence on the structural relationships between the employed variables. The fact that the study pointed out early, weak, and limited evidence of statistical mediation among these variables itself is not enough to consider it to be an example of bad science. A plethora of studies used similar statistics (some of which inspired our work) and provided in the same way evidence of mediated effects. Indeed, all the studies providing early evidence of a relationship between variables without cross-validating it might be assumed to be capitalizing on chance, and could be pointed to as examples of bad science. This is a radical—somewhat Manichean—vision of research, in our opinion. Certainly, all statistical models are approximations. Anyway, following the famous statistician George Box, we could affirm that “all models are wrong, but some are useful”. In this report, the potential usefulness of the studied model of mediation in driving research was highlighted by providing possible implications.

## 3. How Do These Results Advance Our Understanding of Brain Function and/or Human Behavior?

Spatial perception, memory, and mental representation are a series of connected cognitive skills useful for driving. Specifically, both egocentric (self-to-object) and allocentric (object-to-object) spatial representations are assumed to be crucial when navigating by car by supporting the management of the vehicle as, for example, when we turn at an intersection, or we drive through a roundabout. This occurs thanks to the flexible alternation between the two kinds of spatial representation in a process known as spatial updating, underlying navigation by walking as well as by driving [14,15,16]. Specific neural correlates of spatial updating have been previously investigated by other works [14]; the study in question was not focused on neural processes. Anyway, our first study highlighted (i) a significant predictive effect of mental rotation skills on both reaction speed and resilience of attention, and (ii) a significant effect of perspective taking on perceptual speed. Considering the above, it seemed likely that both mental rotation and perspective taking (as measures of object- and self-based spatial transformation, respectively) were playing a mediation role between the cognitive functioning and the driving efficiency. The hypothesis of the second study implied that imagined dynamic spatial transformations (which depend on the individual’s general cognitive functioning) have an influence on driving performance by facilitating both the visualization of the location of stimuli and the anticipation of their movement in the driving scenarios. This influence was expected, being founded on the prerequisites of fitness to drive that underlie the safe management of the vehicle during complex maneuvers. In this way, the study in question was aimed to reduce a knowledge gap on the role of basic spatial transformation skills in the relationship between cognition and driving behaviors. Significant results were found for the indirect effects of cognitive functioning through both mental rotation and perspective-taking skills on the resilience of attention and perceptual speed, respectively. Results suggest that the relationship between cognitive functioning and driving abilities seems to be mainly explained by individual differences in spatial transformations skills. Theoretically, results imply that the specific processes of encoding, the imagined rotation of objects [15], and spatial orientation support drivers’ abilities to manage their vehicles in stressful traffic situations and to quickly detect visual stimuli from the traffic scenarios.

Undoubtedly, this suggests the key role of spatial transformation skills in the execution of complex driving behaviors. Moreover, by relating both spatial transformation skills and cognitive functioning with driving measures, these results may be useful for the assessment of fitness to drive, the training of impaired driving abilities, and in studying the interaction between the driver and the in-vehicle systems. Since spatial transformation skills are key cognitive components of fitness to drive, these measures should be included in the assessment, and it could be the target of training programs for driving capability. In-vehicle systems aid the driver during navigation and in detecting visual scenarios beyond their own visual field. Advanced driver-assistance systems support and facilitate complex maneuvers, such as overtaking or parking. Sensors, cameras, and monitors detect and display the driving scenario out of the driver’s visual field, helping to minimize errors during the maneuver. Mental rotation and perspective-taking abilities are involved in both the visualization and decoding of the spatial relations provided by these devices from the displayed perspective. The study of spatial transformation skills may help researchers to better understand the driver’s interaction with these devices. However, further research is needed to assess whether spatial transformation skills contribute to the readability of the spatial information provided by these systems.

Following Kelly et al., that the MoCA and spatial skills correlate to some degree with the significant indirect effects of spatial skills is not surprising. Certainly, the results could be explained with reference to shared visuospatial processes that are involved in the employed measures. This explanation was put forward frequently in the discussion of the results. Anyway, what surprises us is that both the spatial skills seem to work better if they are considered to be mediators in the model, rather than if they are considered to be predictors. It should be noted that among the theoretical assumptions of the statistical mediation, the association between the independent variable and the mediator is mandatory [16,17]. As reported in Table 1 of the study, there were no issues of collinearity for correlations between the MoCA and all the other variables. However, if there had been a complete overlap of common visuospatial processes shared by the spatial skills and the MoCA, no significant difference would be supposed to emerge between the model with direct effects and the mediation model. Instead, the relationship between global cognitive functioning and driving prerequisites was stronger if studied without the mediation of the spatial skills. Conversely, in the mediation model, the influence of cognition on driving prerequisites was reduced, since it was explained partially by spatial skills. Therefore, the mediated model provided a more exhaustive explanation of fitness-to-drive variability than the single direct effect of the MoCA. If the overlap between the measures of the MoCA with those of spatial transformation seems so obvious, why, then, did considering spatial skills to be mediators provide stronger results? In other words, if spatial skills were superimposed to the predictor, why did they show better fit if treated as mediators rather than as predictors?

In fact, what we found suggests that the relationship between global cognitive functioning and certain driving abilities is mostly a matter of spatial transformation skills.

As stated above, the research project including the commented-upon study/studies also involves the investigations on a clinical sample of drivers. For this reason, the MoCA test was employed among the test set. Moreover, the employment of the MoCA as a measure of general cognitive efficiency needs further clarifications. First, the considered non-pathological range for the MoCA total score included 12 points (i.e., from 18 to 30). Second, the distribution of the MoCA scores deviated from normality, but measures of skewness (|0.195|) and kurtosis (|0.579|) did not exceed critical thresholds [18]. Both these points suggested that the observed variability of the MoCA total score in the sample was adequate to potentially explain the variability of other measures. In addition, this variability showed to be enough to discriminate between different levels of cognitive functioning within the normality range. Given these conditions, we just wanted to consider those individual differences included in the range of the normal cognitive functioning (according to the Italian cut-off). Certainly, the considered non-pathological range of the MoCA total score cannot exclude a person with some degree of cognitive decline, especially for older participants. Indeed, the considered cut-off score was found to discriminate probable cognitive impairment and not probable dementia (i.e., cut-off: 14) [19]. However, other studies validating the MoCA on a southern Italian sample found a lower cut-off score than that used in our study (e.g., 15, 5) [20]. As suggested by Kelly et al., the MoCA test is a screening measure of global cognitive impairment rather than global cognitive functioning. We agree with them. A strong correlation coefficient between the MoCA and age indirectly suggests the usefulness of the MoCA as a measure of cognitive impairment. Although a significant correlation between age and the MoCA was observed, the coefficient of −0.445 suggested a moderate and negative association between these variables. Anyway, it was likely that a source of individual difference in global cognitive functioning was detected in the considered non-pathological range. To this end, individual differences in global cognition, measured with the MoCA score, were considered to be predictors in the studied model.

For the same purposes, the MoCA total score had been used as a measure of “global cognitive functioning” or “cognitive screening” in other studies [21], including healthy young [22], healthy adults [21,22], and healthy elderly [23] participants. Some of these studies provided evidence of the utility of the MoCA in predicting the performance of healthy participants in cognitive tasks. Moreover, other instruments of mental status similar to the MoCA have been employed as measures of cognitive functioning in the study of the influence of cognition on fitness to drive in samples of young, adult, and elderly drivers [12,13,24]. It should be noted that the aims of the studies were far away from making assumption on cognitive decline. The usefulness of the MoCA indicated by Kelly et al. in detecting cognitive impairment assumes the original diagnostic use that was not in line with the aims of our study.

The case of the apparent association between two variables that is reversed when the analysis is performed by considering a third confounding variable is known as Simpson’s paradox [25]. The risk of not consider a spurious relationship that involves one or more confounding variables that cause the studied correlation is a threat for any nonexperimental cross-sectional study [26]. The Simpson’s effect regards qualitative changes in marginal correlations between categorical variables, but it can occur even between continuous variables. Some methods and good practices have been discussed to avoid the risk of this paradox. For example, detecting early potential confounding variables through an exhaustive literature review, then identifying relevant confounding variables through pilot studies, and finally deciding upon an appropriate study design by balancing groups on the confounding variables has been suggested [27]. Simpson’s paradox “could not arise” if participants are equally distributed in groups based on the confounding variable; thus, some statistical techniques have been suggested to produce balanced groups (e.g., simple randomization, randomized block design, and minimization) [27]. These practices imply that large sample sizes produce proportional distributions [28], their accuracy is influenced by the number of confounding variables [27], and they are vulnerable to the presence of interaction between confounding variables [29]. Anyway, two elements are needed to allow the occurrence of this paradox: a neglected confounding variable and unequal distributions of the confounding variable among groups. Considering the study in question, we agree with Kelly et al. on the potential influence of several confounding variables, such as physical or mental activity, which could have explained the significant effects that were found. Particularly, we assumed that the driving frequency was one crucial variable that could explain the studied relationships. The potential confounding effects due to driving frequency/experience have not been observed in the path analysis, but by enrolling only participants who drove at least one time in the week, it was likely that the variability attributable to that variable has at least been reduced. To eliminate the risk of Simpson’s paradox by controlling for any potential confounding variable that could influence a studied relationship is a utopia.

Finally, in response to questions of Kelly et al., it could be useful to clarify that (i) the MoCA was not used to define clinical samples, but as measure of general cognitive functioning, and (ii) the study did not aim to investigate comparisons between healthy and cognitively impaired people. Of course, the employment of the MoCA in the assessment of FtD would be better suited for studying older drivers’ driving abilities than those of the young and adults. In these studies, we were interested in investigating the driving abilities of drivers in age stages which preceded the explicit cognitive impairment. The study of driving fitness in participants with cognitive impairment remains an open issue in driving research that needs exhaustive investigations, but it was not the aim of our paper(s).

## Figures and Tables

**Table 1:** Enrolment criteria for participants involved in both study 1 (Tinella et al., 2020) [2] and study 2 (Tinella et al., 2021) [3].

	Enrollment Criteria		
		Study 1	Study 2
1.	Have Italian as their mother tongue	✓	✓
2.	Hold a valid current driver’s license, provisional or above	✓	✓
3.	Have normal or corrected to normal vision	✓	✓
4.	Not being or had been a professional driver (e.g., taxi driver, truck driver, transporter on delivery, etc.)	✓	✓
5.	Have driven at least one time within the last	month	week
6.	Be in a healthy cognitive state (MoCA inclusion cut-off above 17)	-	✓

## References

[1] Kelly R.E., Ahmed A.O., Hoptman M.J. (2022). What Do These Findings Tell Us? Comment on Tinella et al. Cognitive Efficiency and Fitness-to-Drive along the Lifespan: The Mediation Effect of Visuospatial Transformations. *Brain Sci.* 2021, *11*, 1028. Brain Sci..

[2] Tinella L., Lopez A., Caffò A.O., Grattagliano I., Bosco A. (2020). Spatial mental transformation skills discriminate fitness to drive in young and old adults. Front. Psychol..

[3] Tinella L., Lopez A., Caffò A.O., Nardulli F., Grattagliano I., Bosco A. (2021). Cognitive Efficiency and Fitness-to-Drive along the Lifespan: The Mediation Effect of Visuospatial Transformations. Brain Sci..

[4] Kerr N.L. (1998). HARKing: Hypothesizing after the results are known. Pers. Soc. Psychol. Rev..

[5] Rubin M. (2017). When Does HARKing Hurt? Identifying When Different Types of Undisclosed Post Hoc Hypothesizing Harm Scientific Progress. Rev. Gen. Psychol..

[6] Nori R., Palmiero M., Bocchi A., Giannini A.M., Piccardi L. (2020). The specific role of spatial orientation skills in predicting driving behaviour. Transp. Res. Part F.

[7] Maycock G. (2001). Forecasting older driver accidents and casualties. Road Saf. Res. Rep..

[8] Raitanen T., Törmäkangas T., Mollenkopf H., Marcellini F. (2003). Why do older drivers reduce driving? Findings from three European countries. Transp. Res. Part F.

[9] Jones V., Johnson R., Borkoski C., Gielen A., Rebok G. (2020). Perceived Social Support Differences between Male and Female Older Adults Who Have Reduced Driving. AAA LongROAD Study. (Research Brief).

[10] Mitchell C.G. (2008). The licensing of older drivers in Europe—A case study. Traffic Inj. Prev..

[11] Vernon E.K., Babulal G.M., Head D., Carr D., Ghoshal N., Barco P.P., Morris J.C., Roe C.M. (2015). Adults Aged 65 and Older Use Potentially Distracting Electronic Devices While Driving. J. Am. Ger. Soc..

[12] Ledger S., Bennett J.M., Chekaluk E., Batchelor J. (2019). Cognitive function and driving: Important for young and old alike. Transp. Res. Part F.

[13] Ledger S., Bennett J.M., Chekaluk E., Batchelor J., Di Meco A. (2019). Cognitive function and driving in middle adulthood: Does age matter?. Transp. Res. Part F.

[14] Wolbers T., Hegarty M., Büchel C., Loomis J.M. (2008). Spatial updating: How the brain keeps track of changing object locations during observer motion. Nat. Neurosci..

[15] Jansen P., Render A., Scheer C., Siebertz M. (2020). Mental rotation with abstract and embodied objects as stimuli: Evidence from event-related potential (ERP). Exp. Brain Res..

[16] Baron R.M., Kenny D.A. (1986). The moderator-mediator variable distinction in social psychological research: Conceptual, strategic, and statistical considerations. J. Pers. Soc. Psychol..

[17] Hayes A.F., Scharkow M. (2013). The relative trustworthiness of inferential tests of the indirect effect in statistical mediation analysis: Does method really matter?. Psych. Sci..

[18] West S.G., Finch J.F., Curran P.J., Hoyle R.H. (1995). Structural equation models with nonnormal variables: Problems and remedies. Structural Equation Modeling: Concepts, Issues, and Applications.

[19] Bosco A., Spano G., Caffò A.O., Lopez A., Grattagliano I., Saracino G., Pinto K., Hoogeveen F., Lancioni G.E. (2017). Italians do it worse. Montreal Cognitive Assessment (MoCA) optimal cut-off scores for people with probable Alzheimer’s disease and with probable cognitive impairment. Aging Clin. Exp. Res..

[20] Pirrotta F., Timpano F., Bonanno L., Nunnari D., Marino S., Bramanti P., Lanzafame P. (2015). Italian validation of Montreal cognitive assessment. Eur. J. Psychol. Assess..

[21] Bottiroli S., Tassorelli C., LaMonica M., Zucchella C., Cavallini E., Bernini S., Sinforiani E., Pazzi S., Cristiani P., Vecchi T. (2017). Smart aging platform for evaluating cognitive functions in aging: A comparison with the MoCA in a normal population. Front. Aging Neurosci..

[22] Pike N.A., Poulsen M.K., Woo M.A. (2017). Validity of the Montreal cognitive assessment screener in adolescents and young adults with and without congenital heart disease. Nurs. Res..

[23] Kopecek M., Bezdicek O., Sulc Z., Lukavsky J., Stepankova H. (2017). Montreal Cognitive Assessment and Mini-Mental State Examination reliable change indices in healthy older adults. Int. J. Ger. Psychol..

[24] Kwok J.C.W., Gélinas I., Benoit D., Chilingaryan G. (2015). Predictive validity of the Montreal Cognitive Assessment (MoCA) as a screening tool for on-road driving performance. Br. J. Occup. Ther..

[25] Simpson E. (1951). The interpretation of interaction in contingency tables. J. R. Stat. Soc. Ser. B (Methodol.).

[26] Salkind N.J. (2010). Encyclopedia of Research Design.

[27] Ameringer S., Serlin R.C., Ward S. (2009). Simpson’s paradox and experimental research. Nurs. Res..

[28] Hsu L.M. (1989). Random sampling, randomization, and equivalence of contrasted groups in psychotherapy outcome research. J. Cons. Clin. Psychol..

[29] Tu D., Shalay K., Pater J. (2000). Adjustment of treatment effect for covariates in clinical trials: Statistical and regulatory issues. Drug Inf. J..

